# Genome-Wide Interaction Study of Omega-3 PUFAs and Other Fatty Acids on Inflammatory Biomarkers of Cardiovascular Health in the Framingham Heart Study

**DOI:** 10.3390/nu9080900

**Published:** 2017-08-18

**Authors:** Jenna Veenstra, Anya Kalsbeek, Jason Westra, Craig Disselkoen, Caren E. Smith, Nathan Tintle

**Affiliations:** 1 Department of Mathematics, Statistics, and Computer Science, Dordt College, Sioux Center, IA 51250, USA; jnnvnstr@dordt.edu (J.V.); nyklsbk@dordt.edu (A.K.); westrajason@hotmail.com (J.W.); cdisselk@eng.ucsd.edu (C.D.); 2 Jean Mayer USDA Human Nutrition Research Center on Aging, Tufts University, Boston, MA 02153, USA; Caren.Smith@tufts.edu

**Keywords:** fatty acids, omega-3, alpha linoleic acid, docosahexaenoic acid, inflammatory biomarkers, cardiovascular disease

## Abstract

Numerous genetic loci have been identified as being associated with circulating fatty acid (FA) levels and/or inflammatory biomarkers of cardiovascular health (e.g., C-reactive protein). Recently, using red blood cell (RBC) FA data from the Framingham Offspring Study, we conducted a genome-wide association study of over 2.5 million single nucleotide polymorphisms (SNPs) and 22 RBC FAs (and associated ratios), including the four Omega-3 FAs (ALA, DHA, DPA, and EPA). Our analyses identified numerous causal loci. In this manuscript, we investigate the extent to which polyunsaturated fatty acid (PUFA) levels moderate the relationship of genetics to cardiovascular health biomarkers using a genome-wide interaction study approach. In particular, we test for possible gene–FA interactions on 9 inflammatory biomarkers, with 2.5 million SNPs and 12 FAs, including all Omega-3 PUFAs. We identified eighteen novel loci, including loci which demonstrate strong evidence of modifying the impact of heritable genetics on biomarker levels, and subsequently cardiovascular health. The identified genes provide increased clarity on the biological functioning and role of Omega-3 PUFAs, as well as other common fatty acids, in cardiovascular health, and suggest numerous candidate loci for future replication and biological characterization.

## 1. Introduction

Genome-wide association studies (GWAS) have been previously used to identify common single nucleotide polymorphisms (SNPs) associated with fatty acid (FA) levels [1,2].There have also been studies showing correlations between FA levels, specifically Omega-3 and Omega-6 FAs, and a variety of disease phenotypes and risk factors, including total mortality [3], acute coronary syndrome [4,5], serum lipid levels [6], cognitive function [7], brain size [8,9], and inflammatory biomarkers [10]. Such biomarkers, including C-reactive protein, are known risk factors of cardiovascular disease [11]. Though studies exist that examine the influence FAs have on inflammation, the understanding of the relationship remains incomplete. In particular, there are very few studies of FA–genetic interactions on inflammatory outcomes. Thus, there is a large gap in our understanding of how and where FAs, specifically Omega-3 and Omega-6 FAs, modulate genetic risk. Thus, little is known as to how genetics might alter the optimal dietary FA intake for the individual.

For example, using data from the Framingham Heart Study, a comprehensive, longitudinal study of cardiovascular health, (a) genes associated with fatty acid levels [2], (b) genes associated with inflammatory biomarkers [12,13,14,15], and (c) the association between fatty acid levels and inflammatory biomarkers have been identified [16]. To date, little work has been done to comprehensively integrate genetic, fatty acid, and inflammatory biomarker data to more precisely articulate the complex relationship between these factors.

Recently, to help elucidate more complex genetic pathways towards disease processes, genome-wide interaction studies (GWIS) have been used to attempt to identify specific lifestyle exposures that may modulate genetic associations with disease outcomes [17]. In the case of FAs and inflammatory biomarkers, testing for potential interactions between SNPs and FAs on biomarker levels may provide a more complete picture as to how an individual’s genetics modulate FA levels leading to differential effects on biomarker levels. 

Here, we report a GWIS exploring the interaction between the relative proportions of twelve FAs with over 2.5 million common (minor allele frequency >1%) SNPs and its relationship with nine inflammatory biomarkers among participants in the Framingham Heart Offspring Study.

## 2. Materials and Methods

### 2.1. Sample

We conducted our analysis on the Offspring Cohort of the Framingham Heart Study (FHS). This sample consists of the children and spouses of the original FHS cohort, who were recruited in 1971. Detailed descriptions of the sample are available elsewhere [6,18,19,20]. The final sample consisted of 2700 individuals as a subset of the Offspring subjects that attended Examination 8 (2005–2008). Written informed consent was provided by all participants, and the Institutional Review Boards at Boston University approved the Framingham data collection protocols, while Dordt College approved the analysis protocol for this manuscript.

### 2.2. Fatty Acids

We chose to use red blood cell (RBC) samples, because they have been shown to be more representative of FA levels in other tissues/cells [21], and with mounting evidence that this fatty acid pool may be less affected by recent fat consumption [3]. The RBC samples were analyzed for glycerophospholipid FA composition using gas chromatography as previously described [6]. We primarily focused on Omega-3 (n3) and Omega-6 (n6) FAs for this study: eicosapentaenoic acid (EPA, n3), docosapentaenoic acid- n3 (DPA, n3), docosahexaenoic acid (DHA, n3), alpha-linoleic acid (ALA, n3), linoleic acid (LA, n6), gamma-linoleic acid (GLA, n6), dihomo-gamma-linoleic acid (DGLA, n6), arachidonic acid (AA, n6), docosapentaenoic acid-n6 (DPA, n6), docosatetranoic acid (DTA, n6), eicosadienoic acid (EDA, n6), and oleic acid (OA, n9). Means and standard deviations (SDs) are provided in Table 1. These twelve FAs were selected because circulating polyunsaturated fatty acids (PUFA) have been shown to be protective against cardiovascular disease (CVD) [22,23], while the health impacts of omega-6 PUFAs are still under debate [24,25]. OA was also included in this study as an exploratory look into the impacts of Omega-9 FAs [26], because it is one of most common FAs (see Table 1), and because we have previously identified a number of genetic variants associated with it [2]. The other ten FAs measured in the FHS that are included in this analysis are palmitic acid, stearic acid, palmitoleic acid, myristic acid, palmitelaidic acid, trans oleic acid, trans linoleic acid, eicosenoic acid, lingnoceric acid, and nervonic acid, and are also included in Table 1.

### 2.3. Biomarkers of Inflammation

Biomarkers were analyzed using commercially available assays by labs affiliated with the Framingham Heart Study. Their collection, storage, distribution, and measurement procedures are publicly available in their FHS Inflammatory Marker Manuals [27]. Nine inflammatory biomarkers were included in this analysis: C-reactive protein (CRP), Intercellular adhesion molecule 1 (ICAM), Cellular adhesion molecule (CAM), Interleukin-6 (IL6), Lipoprotein-Associated Phospholipase A2 (LP-PLA2 activity denoted by “PLAC”), Monocyte chemoattractant protein-1 (MCP1), Osteoprotegerin (OPG), P-Selectin, and Tumor necrosis factor receptor II (TNF). These biomarkers were selected based on their identified association with CVD risk [16,28,29,30,31,32,33,34,35].

### 2.4. Genotype Data

Genotypes were originally measured using the Affymetrix 500K chip as previouslydescribed [2]. We imputed these markers on the University of Michigan imputation server [36] using the HAPMAP 2 reference panel, yielding approximately 2.5 million variants meeting standard quality control criteria and with minor allele frequency (MAF) >1% [37,38]. After imputation, variants were assigned rsIDs and genes based on chromosomal position from build 37 and Ensemble release 75, based on GRCHh37.p13 [39].

### 2.5. Statistical Analysis

Regression was used to predict the log-transformed value of each biomarker. We fit a model as $Biomarker=\beta_{0}+\beta_{1}SNP+\beta_{2}FA+\beta_{3}SNP*FA+Age+Sex$, where SNP takes a value 0, 1, or 2 based on the number of minor alleles present for the individual at the SNP being modeled (or, potentially, a fractional value between 0 and 2 for imputed SNPs). Windsorization was used to reduce the impact of outliers in FAs and biomarkers, by using values four SDs from the mean in cases where observed values were more than four SDs from the mean. The model was fit for each SNP–FA combination separately. Single marker tests were deemed statistically significant when the p-value for the interaction term in the model was less than 5 × 10^–8^, a standard criterion for genome-wide significance [40]. Significance of the interaction term indicates that the interaction between the SNP and FA adds significantly more explanation of biomarker levels than the SNP and FA alone. A follow up analysis fit the model $Biomarker=\beta_{0}+\beta_{1}SNP$ for each SNP in order to evaluate whether an SNP would have been identified as important and related to the biomarker without considering fatty acid values in the analysis. LocusZoom [41] was used to generate figures depicting statistical significance, linkage disequilibrium (LD) structure, and gene locations. Genomic control lambdas (λ_GC_) were estimated and Q-Q plots were computed. λ_GC_ values showed no evidence of overinflation of test statistics [42]. Less than 1% of the sample was dropped from any particular analysis due to missing data (genetic, FA, or biomarker data).

## 3. Results

The clinical characteristics for the FHS Offspring cohort participants have been previously reported along with their FA levels, and so are not described in detail here [2]. Briefly, the mean (SD) age was 66 (9) years, 54% were female, 9% smoked, 49% were treated for hypertension, and 43% were being treated for high cholesterol. Fourteen percent of the sample was diabetic, 11% had coronary heart disease, and 3% had congestive heart failure.

In total, we identified eighteen unique genome-wide significant (*p* < 5 × 10^–8^) SNP by FA interaction terms (Table S1). No single SNP was significant for more than one biomarker. Six of the nine biomarkers had significant interactions (all except OPG, PLAC, and P-selectin). Seven of the FAs under consideration in our analysis had no interactions with any SNPs reach the genome-wide significance level (DPA(N3), EPA, AA, DGLA, DTA, GLA, and LA), but the other five FAs had at least one interaction with a SNP reach genome-wide significance, including two Omega-3 FAs (ALA and DHA). In subsequent discussion about the result, the biomarker levels are reported as pg/mL, and the FA levels are reported as percent composition.

We have summarized these results by focusing on distinct regions (1 MB or smaller) containing the significant interactions. Table 2 provides an overview of the eight 1 MB regions covering the 18 identified SNPs, summarizing location information, the number of significantly associated interactions, references to prior literature about the functionality of the region, and a listing of the associated biomarkers.

### 3.1. Chromosome 1: CHRM3

**Figure 1:**
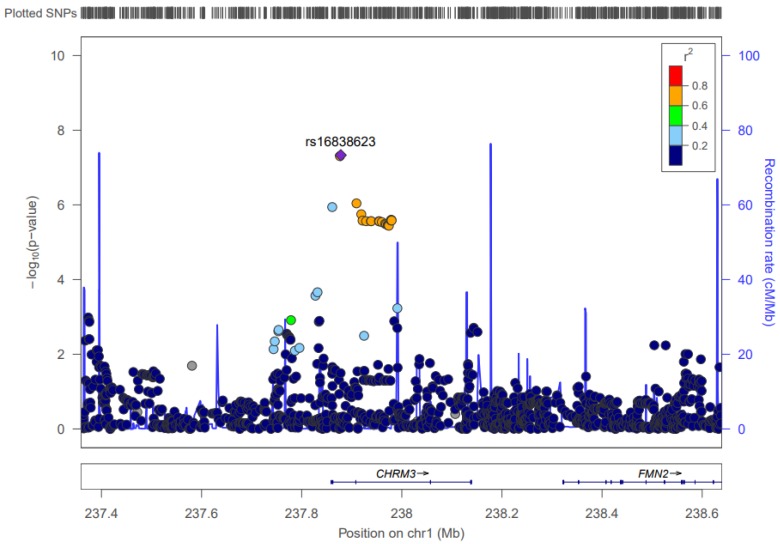
LocusZoom Plot for *CHRM3*. This figure shows the linkage disequilibrium and significance pattern of SNPs in and around the *CHRM3* gene.

Two significant interactions contain SNPs located within the *CHRM3* gene (239,549,865 to 240,078,750). These SNPs (rs16838623 and rs16832149), when interacting with ALA, become significantly associated with the IL6 biomarker, with *p*-values of 4.66 × 10^–8^ and 4.95 × 10^–8^, respectively. The average effect allele frequency for the two SNPs is 0.0225, and both SNP × FA interactions have an estimated slope of approximately 0.9. This means that for every additional copy of either of the effect alleles, IL6 log-transformed levels increase by about 0.9 due to a higher ALA impact. See Figure 1 for an illustration of the LD structure and significance of SNPs in the CHRM3 gene.

**Figure 2:**
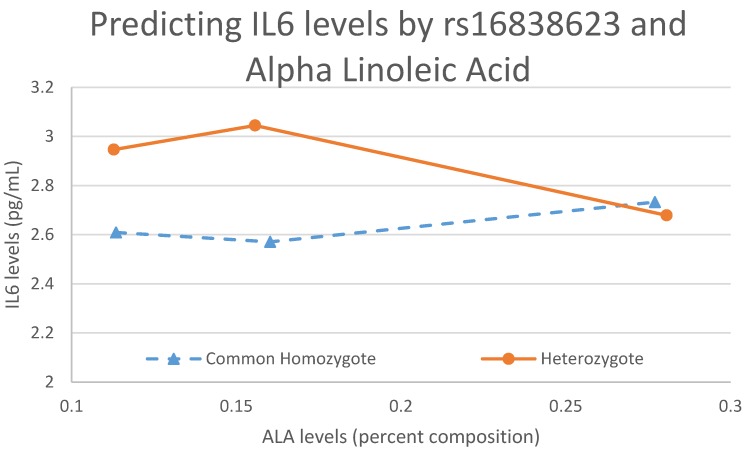
An interaction plot between ALA and rs16838623 predicting IL6 levels. This figure shows the modulating effects the genotype has on FAs and the effect that interaction has on biomarker levels. The rare homozygote is not included in this plot because, in our sample, there is only one individual with that genotype.

Figure 2 demonstrates that when a significant interaction exists, an individual’s genotype has modulating effects on FA levels, which taken together predict the level of a specific biomarker, in this case IL6. Figure 2 shows that one copy of the effect allele interacts with an increasing level of ALA to ultimately decrease the IL6 levels. The common homozygote trend is clearly the opposite of the heterozygote, exemplifying an interaction.

### 3.2. Chromosome 2: RPL7P61

There are four significant interactions whose SNPs are near the *RPL7P61* gene, which is located from bp 164,008,761 to 164,009,817 on chromosome 2. When interacting with DHA, these SNPs are associated with IL6, giving *p*-values that range from 8.88 × 10^–9^ to 3.05 × 10^–9^, with an average effect allele frequency of approximately 0.016. The effect size for the interaction ranges from 3.02 to 3.11, meaning that the impact of DHA on the log-transformed IL6 levels increases by about 3 for every additional copy of the effect allele.

**Figure 3:**
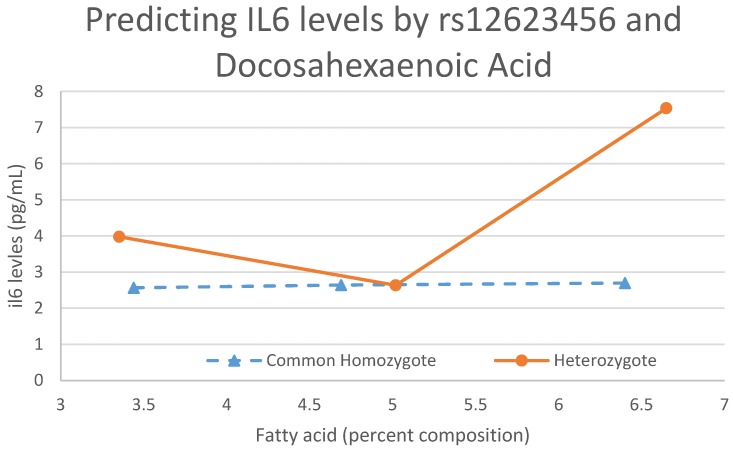
An interaction plot between DHA and rs12623456 predicting IL6 levels. This figure shows the modulating effects the genotype has on FAs and the effect that interaction has on biomarker levels. The rare homozygote is not included in this plot because, in our sample, there are no individuals with that genotype.

Figure 3 shows another interaction between SNP rs12623456 and DHA to predict IL6. In this interaction, the trends are the opposite of what they are for Figure 2. With no copies of the effect allele, there is very little change in IL6 levels as DHA levels increase. As soon as a copy of the allele is added, the increase in FA level corresponds overall to an increase in IL6 levels. However, due to the low effect allele frequency for this SNP (0.0161), the trend for when there are two copies of the effect allele is not attainable with our sample size.

### 3.3. Chromosome 3: RP11-373E16.1

The *RP11-373E16.1* gene, located from 170,371,635 to 170,372,048, contains two SNPs (rs7611820 and rs9856712) that interact with OA to predict MCP1 levels. The *p*-values range from 5.87 × 10^–10^ to 5.25 × 10^–10^. The average effect allele frequency for the two SNPs is approximately 0.072 and the average effect size for the interactions is −1.318, which means that for every additional copy of either effect allele, the impact OA has on log-transformed MCP1 levels decreases by about 1.318.

### 3.4. Chromosome 7: CHCHD3

There are two SNPs in chromosome 7 that, when interacting with DPA(N6), are significantly associated with ICAM, giving p-values of 1.07 × 10^–8^ and 1.00 × 10^–8^ (rs17424227 and rs17424324). Both SNPs are near the *CHCHD3* gene (132,469,629 to 132,469,629). The average effect allele frequency for the two SNPs is 0.116. The impact DPA(N6) has on the log-transformed levels of ICAM decreases by about 0.29 for every additional copy of the effect allele.

### 3.5. Chromosome 13: LOC105370115

One SNP (rs17079653), near the *LOC105370115* gene on chromosome 13, contributes in an interaction with OA to predict TNF levels with a *p*-value of 2.88 × 10^–8^. The effect allele for the SNP is 0.0233, and the effect size for the interaction is −15.49. Thus, for every additional copy of the effect allele, the impact OA has on log transformed TNF levels decreases by 15.49.

### 3.6. Chromosome 14

#### 3.6.1. *LOC105378178*

In chromosome 14, there is one interaction with EDA and the SNP rs7160151 that is significantly associated with CRP at a *p*-value of 2.93 × 10^–8^. The SNP in this interaction is located near the *LOC105378178* gene. The SNP has an effect allele frequency of 0.29, and the effect size for the interaction is 1.19, meaning that for each additional copy of the effect allele, log-transformed CRP levels increase by 1.19 due to the increased impact of EDA.

#### 3.6.2. *CTD-3006G17.2*

**Figure 4:**
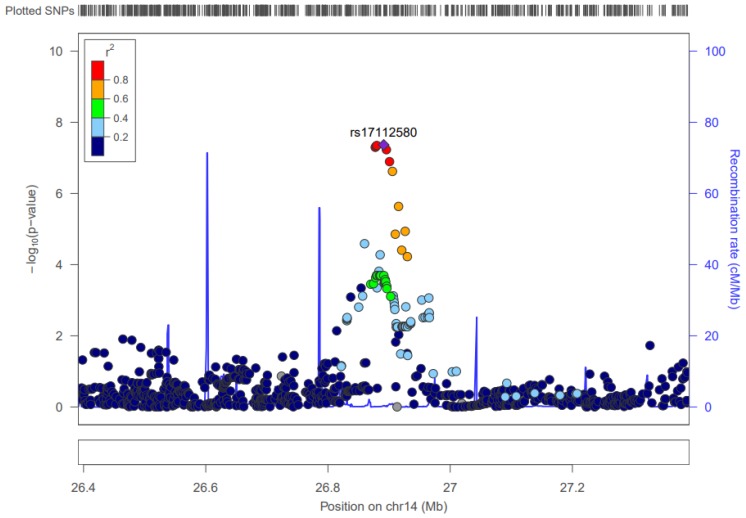
LocusZoom Plot for rs17112580. Note: Start-stop positions for *CTD-3006G17.2* are not depicted in this figure since it is a pseudogene.

There are three SNPs in the *CTD-3006G17.2* gene, which is located from 27,727,923 to 28,314,492, that contribute to a significant interaction. All three interact with OA, and are associated with CAM with *p*-values as low as 4.33 × 10^–8^. Minor allele frequencies are in the 0.25 range for these SNPs. The effect size of the corresponding interactions for the three is around −0.57. For each additional copy of the three alleles, the impact OA has on log-transformed CAM levels decreases by about 0.57. Figure 4 shows the linkage disequilibrium and significance pattern of SNPs in and around the SNP rs17112580.

### 3.7. Chromosome 20: LINC00652

**Figure 5:**
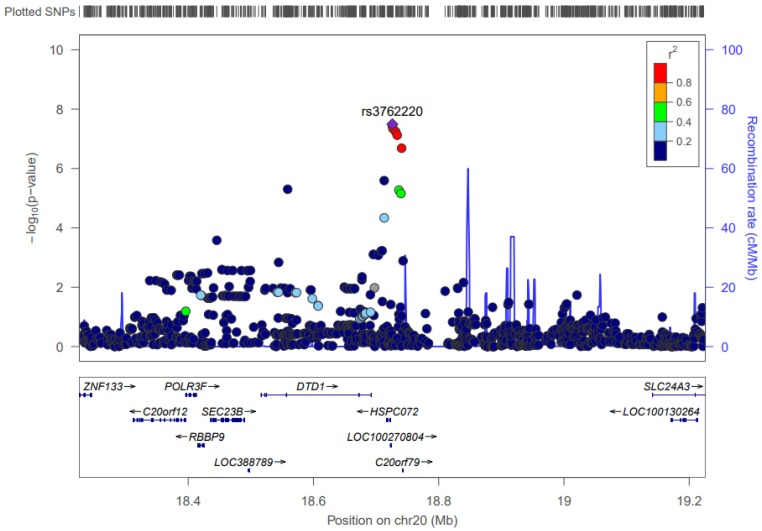
LocusZoom Plot for rs3762220. This figure shows the linkage disequilibrium and significance pattern of SNPs in and around the SNP rs3762220.

The *LINC00652* gene (18,768,295 to 18,774,979) is in close proximity to three SNPs that, when interacting with OA, are associated with the CRP biomarker. These interactions have *p*-values that range from 4.54 × 10^–8^ to 3.23 × 10^–8^. The effect allele frequency for the three corresponding SNPs are all around 0.048. The effect size for the interactions ranges from 5.129 to 5.215, which means that every additional copy of any of the effect alleles increases the impact OA has on log-transformed CRP levels by about 5.15. Figure 5 shows the pattern of significance and LD structure.

## 4. Discussion

In previous analyses on this sample, we did not consider potential moderating effects of SNPs on the associations between fatty acids and inflammatory biomarkers, as we have done here. Notably, none of the SNPs we identified have been previously associated with inflammatory biomarkers or with fatty acid levels. Without including the interaction term in our models, none of the SNPs contributing to the interaction would have been discovered. Our results confirm the utility of interaction studies and provide specific interactions to investigate further.

We focus the remainder of our discussion on the eighteen loci that show strong evidence of an interaction with five separate FAs (DPA(N6), EDA, OA, ALA, and DHA) on inflammatory biomarkers. DPA(N6) and EDA have been somewhat understudied in the literature; however, there is a study that associates increased amounts of long-chain Omega-6 fatty acids with lower risk for CVD, which includes both DPA(N6) and EDA [43], and EDA was observed in vitro to modulate the metabolism of other FAs and therefore the inflammatory response [44]. A recent study indicated that OA plays a protective role against cardiovascular health, and has been shown to improve endothelial dysfunction when inflammatory signals are present, thus stabilizing arteries’/veins’ response to adverse immune response [45,46]. ALA has previously been shown to be associated with CVD risk [47,48]. Finally, DHA is an Omega-3 fatty acid that has been associated with cardiovascular disease risk, and evidence suggests that it has anti-arrhythmic and anti-thrombotic effects when consumed as a dietary supplement in fish oil pills [49].

### 4.1. Docosapentaenoic Acid (Omega-6)

There are two SNPs in chromosome 7 that interact with DPA(N6) to predict ICAM. These SNPs (rs17424324 and rs17424227) are located near the *CHCHD3* gene, which codes for an inner mitochondrial membrane scaffold protein. While *CHCHD3* has no prior GWAS evidence, the protein has been shown to be essential for maintaining crista integrity and mitochondrial function. Crista structure is very important, as its disruption has been implicated in a variety of cardiovascular and neurodegenerative diseases. Thus, the interaction of DPA(N6) and SNPs near *CHCHD3* may have an impact on CVD [50].

### 4.2. Eicosadienoic Acid

A single SNP located in chromosome 14 (rs7160151) showed evidence of interaction with EDA to predict CRP levels. The SNP is located near the *LOC105378178* gene. While no prior GWAS evidence exists revealing variants within *LOC105378178* as associated with cardiometabolic traits, there is evidence of association with a variety of mental disorders, including ADHD [51] andPTSD [52], Otherwise, this gene remains relatively uncharacterized.

### 4.3. Oleic Acid

#### 4.3.1. Chromosome 3

Two interactions contain SNPs located in chromosome 3 that, when interacting with OA, are associated with MCP1 levels. These SNPs (rs7611820 and rs9856712) are located near the *CLDN11* gene. GWAS has shown no evidence of variants within *CLDN11* as associated with cardiometabolic traits. However, the protein CLDN11 codes for is a tight-junction protein, whose deficiency may lead to cell barrier dysfunction in endothelial cells. Such a deficiency is considered the initiating process and pathological basis of CVD in diabetes [53].

#### 4.3.2. Chromosome 13

One SNP in chromosome 13 contributes to an interaction with OA to predict TNF levels. This SNP (rs17079653) is near the *SPATA13* gene or spermatogenesis-associated 13 gene. Prior GWAS has shown that variants in *SPATA13* are associated with psychological disorders, such as depression and alcohol dependence [54]. To date, there are no studies reporting the association of *SPATA13* with CVD risk factors or FA levels.

#### 4.3.3. Chromosome 14

Three interactions, whose SNPs are located in chromosome 14, are associated with CAM levels. The three SNPs (rs17112580, rs992745, and rs7145681) are near the *CTD-3006G17.2*, which is a pseudogene. There is no prior GWAS evidence for variants within *CTD-3006G17.2* being associated with any cardiometabolic traits or other phenotypes.

#### 4.3.4. Chromosome 20

There are three SNPs in chromosome 20 that contribute to interactions with OA that are associated with CRP levels. The SNPs (rs3762220, rs3827974, and rs3762221) are found near the *DTD1* gene, which codes for a protein associated with the initiation of DNA replication. There is not any prior GWAS evidence for variants in *DTD1* being associated with cardiometabolic traits. There is evidence, however, that this gene is associated with higher levels of DHA as well as with generally higher Omega-3 and Omega-6 FA levels [55]. *DTD1* also may be a risk factor for aspirin-intolerant asthma through a mechanism that promotes a pro-inflammatory phenotype [56].

### 4.4. Alpha Linoleic Acid

There are two interactions to which ALA contributes in chromosome 1 on IL6. These interactions are both in the *CHRM3* (acetylcholine receptor M3) gene, and predict the IL6 biomarker. Prior evidence shows that variants in the *CHRM3* gene are associated with hypertension [57], obesity-related traits [58], and platelet count [59]. All three variables involved in this interaction (ALA, IL6, and *CHRM3*) have previously been shown to be associated with CVD risk [60,61,62]. ALA levels have also been shown to be associated with hypertension [48,63]. Thus, *CHRM3*, as a receptor known to modulate the production of cytokines, is a strong candidate for future exploration in the prediction of CVD risk.

### 4.5. Docosahexaenoic Acid

Four SNPs in chromosome 2 contribute to significant interactions with DHA to predict IL6 levels. These SNPs are located near the *RPL7P61* gene, which is a ribosomal protein pseudogene. Prior genome-wide association studies have identified variants in *RPL7P61* as associated with phospholipid levels [64] as well as breast cancer survival [65].

### 4.6. Limitations

While our analysis identified eighteen interactions between genetic loci and FAs to be associated with biomarker levels, some limitations of our analysis are worth noting. First, our results may be limited by the low levels of these FAs in the blood. Also, the lack of additional covariates other than age and sex may affect the results of our analysis. Further work is needed to develop models that leverage various covariates, including dietary covariates (e.g., consumption and supplementation), that could mask the impact of some SNPs in our current analysis. Second, our analysis did not look at all FAs or FA ratios. Investigating interactions between SNPs and FA ratios may give a clearer picture as to how a genotype modulates FA conversion signifying a few metabolism pathways. We note that this analysis only analyzed RBC FAs. While RBC FAs may have some advantages (e.g., stability over time), other FA samples, including circulating free FAs, may reveal different results [66]. Also, our results are limited to the effects these FAs and their interactions with SNPs have on our nine known inflammatory biomarkers. Further research is necessary to further investigate additional inflammatory biomarkers that may have a more direct relationship with FA levels, as well as additional FAs (e.g., saturated FAs). Next, to date, many of the regions around the loci for the significant interactions are not well understood. This suggests the need for replication, which could lead to novel biological understanding if the findings are replicable in other samples. Finally, our focus on common variants (vs. rare) and linear relationships (vs. non-linear) between SNP × FA interactions and biomarker levels represents one of many models. These numerous other models are worth exploring in subsequent analyses.

## 5. Conclusions

Genome-wide interaction studies are becoming a more widely used method to provide valuable evidence of potential gene–environment interactions’ contributions towards phenotypes of interest. Fatty acid research, especially with Omega-3 FAs, has shown the importance their contribution has on the health of an individual. The interactions identified in this analysis provide more information about specific genes that will contribute to our understanding both of how these genes modulate FA levels, and of how, together, genes and environment affect an individual’s phenotype. Wet-lab exploration of these interactions will likely contribute greatly to the understanding of these effects. Using a meta-analysis to examine larger sample sizes and sequencing data to explore the potential contribution of rare variants to these interactions are also worthy goals in the future.

## Figures and Tables

**Table 1:** Summary of the 12 red blood cell (RBC) fatty acids (FAs) measured by the Framingham Heart Study (FHS) and considered in our analysis.

Fatty Acid	Mean (Percent Composition)	SD
Oleic acid (OA)	13.900%	1.030%
Eicosadienoic acid (EDA)	0.278%	0.046%
Gamma-linoleic acid (GLA)	0.083%	0.072%
Alpha-linoleic acid (ALA)	0.184%	0.098%
Linoleic acid (LA)	11.100%	1.700%
Dihomo-gamma-linoleic acid (DGLA)	1.596%	0.359%
Arachidonic acid (AA)	16.800%	1.600%
Eicosapentaenoic acid (EPA)	0.732%	0.447%
Docosatetranoic acid (DTA)	3.790%	0.826%
Docosapentaenoic acid-*n*-6 (DPA_N6)	0.661%	0.189%
Docosapentaenoic acid *n*-3 (DPA_N3)	2.750%	0.453%
Docosahexaenoic acid (DHA)	4.840%	1.360%

The 10 FAs not analyzed here comprise the additional 55% of fatty acid levels (%, standard deviation (SD)): myristic acid (0.306, 0.08), palmitic acid (21.270, 1.248), palmitelaidic acid (0.167, 0.047), palmitoleic acid (0.359, 0.194), stearic acid (18.108, 0.947), trans oleic acid (1.649, 0.55), trans linoleic acid (0.253, 0.084), eicosenoic acid (0.277, 0.109), lingnoceric acid (0.732, 0.447), and nervonic acid (0.445, 0.151). The mean and standard deviation of percent composition are provided for the *n* = 2703 individuals.

**Table 2:** Summary of eight regions (1MB or less) of significant SNP × FA interactions.

Chr	Region	Sig SNPs	Biomarker	Location (bp)	Smallest Int. *p*-Value (rsid#:FA)	Genes Containing/Near SNPs	Previous Cardiometabolic Trait Evidence	EAF	Significant Without Interaction
1	239,809,739–239,811,390	2	IL6	239,811,390	4.66 × 10^–^^8^ (rs16838623:ALA)	*CHRM3, LOC105373225*	Hypertension [28]	0.0222	No
2	163,855,536–164,056,447	4	IL6	164,019,142	3.05 × 10^–^^9^ (rs12623456:DHA)	*RPL7P61*	None	0.0161	No
3	170,371,857–170,376,150	2	MCP1	170,371,857	5.25 × 10^–^^10^ (rs7611820:OA)	*RP11-373E16.1 CLDN11, LOC101928583, RPL28P1*	None	0.0724	No
7	132,794,130–132,796,323	2	ICAM	132,796,323	1.00 × 10^–^^8^ (rs17424324:DPA_N6)	*LOC105375512, CHCHD3*	None	0.116	No
13	24,533,606	1	TNF	24,533,606	2.88 × 10^–^^8^ (rs17079653:OA)	*LOC105370115, ANKRD20A19P, SPATA13*	None	0.0233	No
14	49,803,164	1	CRP	49,803,164	2.93 × 10^–^^8^ (rs7160151:EDA)	*LOC105378178*	None	0.29	No
14	27,808,931–27,821,399	3	CAM	27,821,399	4.33 × 10^–^^8^ (rs17112580:OA)	*CTD-3006G17.2, LOC728755*	None	0.249	No
20	18,777,980–18,778,844	3	CRP	18,777,980	3.23 × 10^–^^8^ (rs3762220:OA)	*LOC100270804, LINC00652, LOC107985399, EEF1A1P34, DTD1, C20orf78*	None	0.0484	No

SNP, single nucleotide polymorphism; EAF, effect allele frequency. The genes that the significant interaction SNP is in or near (50 KB pair range on each side) is reported. Prior GWAS evidence is reported based on a search at http://www.ebi.ac.uk/gwas/. The column “Significant without interaction” indicates whether the SNP alone would have reached genome-wide significance if the FA interaction term was not in the model.

## Supplementary Materials

The following are available online at www.mdpi.com/2072-6643/9/8/900/s1, Table S1: Summary of eighteen significant interactions.
